# Surgical Timing in Relation to Visual Dysfunction in Skull Base Meningiomas Involving the Anterior Visual Pathway: A Propensity Score-Matched Analysis

**DOI:** 10.3390/cancers18203324

**Published:** 2026-10-15

**Authors:** Takakazu Kawamata, Seiichiro Eguchi, Shuhei Morita, Koji Yamaguchi, Kosaku Amano

**Affiliations:** Department of Neurosurgery, Tokyo Women’s Medical University, 8-1 Kawada-cho, Shinjuku-ku, Tokyo 162-8666, Japan; eguchi.seiichiro@twmu.ac.jp (S.E.); morita.shuhei@twmu.ac.jp (S.M.); yamaguchi.koji@twmu.ac.jp (K.Y.); kamano@twmu.ac.jp (K.A.)

**Keywords:** anterior visual pathway, asymptomatic meningioma, optic nerve, propensity score matching, skull base meningioma, surgical timing, visual outcome

## Abstract

Background/Objectives: Observation is generally recommended for asymptomatic meningiomas, but evidence from incidental meningiomas is derived largely from heterogeneous intracranial locations and may not directly apply to tumors involving the anterior visual pathway. We investigated whether preoperative visual status remained associated with postoperative visual function after adjustment for baseline clinical and anatomical differences in this location-specific subgroup. Methods: We retrospectively reviewed 146 patients who underwent resection of skull base meningiomas contacting or compressing the optic nerve and/or chiasm. Preoperative visual status was categorized by comprehensive ophthalmologic evaluation as normal visual function or visual dysfunction. The primary outcome was postoperative normal visual function, defined as preservation of normal vision in patients with normal preoperative visual function and complete recovery in those with preoperative visual dysfunction. Multivariable logistic regression was performed, and propensity score matching was conducted using age, sex, tumor volume, tumor laterality, and optic canal involvement. Results: Propensity score matching yielded 48 matched pairs with adequate balance across all measured covariates (all standardized mean differences < 0.1). Postoperative normal visual function was preserved or achieved in 95.8% of patients with normal preoperative visual function versus 31.3% of those with preoperative visual dysfunction (exact McNemar *p* < 0.001). Favorable visual outcome occurred in 95.8% versus 77.1%, respectively (*p* = 0.0225). Conclusions: Among surgically treated patients, normal preoperative visual function remained strongly associated with postoperative normal visual function after adjustment for measured clinical and anatomical differences. These findings do not establish that earlier surgery improves visual outcomes or define an optimal surgical timing strategy, but suggest that preoperative visual status and tumor anatomy should be considered in individualized management decisions.

## Simple Summary

Most incidentally detected meningiomas are managed with regular imaging, but tumors that already contact or compress the optic nerves or optic chiasm may require separate consideration. We studied 146 patients who underwent surgery for such skull base meningiomas. To reduce the influence of pre-existing clinical and anatomical differences, we used propensity score matching to create 48 closely balanced patient pairs based on age, sex, tumor size, tumor location, and optic canal involvement. Even after this matching, postoperative normal visual function was substantially more frequent in patients whose visual function had been normal before surgery than in those with preoperative visual dysfunction. These findings do not indicate that all asymptomatic meningiomas should be treated surgically. Rather, they provide quantitative evidence that meningiomas directly involving the anterior visual pathway may warrant location-specific consideration when determining surgical timing.

## 1. Introduction

Skull base meningiomas involving the anterior visual pathway present a distinct management challenge because even relatively small tumors can cause visual impairment through direct compression of the optic nerves or chiasm. Unlike meningiomas at many other intracranial locations, tumor size alone may therefore be insufficient to determine the need for intervention in this anatomically vulnerable region [1].

For incidentally detected asymptomatic meningiomas, observation with serial imaging is generally recommended [2,3,4,5,6]. Longitudinal studies and systematic reviews have characterized the variable natural history of these tumors and form much of the evidence base for surveillance strategies [7,8,9,10]. However, the evidence supporting this approach is derived largely from cohorts that include tumors from heterogeneous intracranial locations and rarely evaluate meningiomas directly involving the anterior visual pathway as a distinct subgroup. Consequently, the general management paradigm for incidental meningiomas may not be directly applicable to tumors in which further growth could compromise visual function.

This issue is particularly relevant in Japan, where brain health screening programs (“Brain Dock”) frequently detect asymptomatic intracranial lesions [11]. The Japanese Brain Dock Guidelines recommend prophylactic surgical resection for medial sphenoid wing meningiomas because of the risk of visual deterioration [12]. However, evidence supporting intervention before objective visual dysfunction develops remains limited. Once visual impairment develops, postoperative recovery may be incomplete [1,13,14,15,16], emphasizing the need to clarify whether surgical timing should be considered differently for tumors involving the anterior visual pathway.

Few studies have directly compared visual outcomes between patients with normal preoperative visual function and those with preoperative visual dysfunction among meningiomas involving the anterior visual pathway. Sturiale et al. [16] reported better postoperative visual outcomes in asymptomatic patients, but adjusted comparisons accounting for baseline clinical and anatomical differences were not performed. In particular, factors such as tumor volume and optic canal involvement may differ between patients with and without preoperative visual dysfunction and confound comparisons of postoperative outcomes.

We therefore investigated surgical timing in patients with skull base meningiomas directly contacting or compressing the anterior visual pathway. Multivariable logistic regression and propensity score matching (PSM) were used to account for baseline differences between patients with and without preoperative visual dysfunction. We hypothesized that preservation of normal visual function would remain associated with surgery performed before objective visual dysfunction developed after adjustment for these differences, supporting a location-specific approach to surgical timing for meningiomas involving the anterior visual pathway.

## 2. Materials and Methods

### 2.1. Ethical Approval

This study was approved by the Institutional Review Board of Tokyo Women’s Medical University (Approval No. 2021-0220) and was conducted in accordance with the Declaration of Helsinki. The requirement for written informed consent was waived by the Institutional Review Board because of the retrospective nature of the study, and patients were provided the opportunity to opt out of the use of their clinical data for research.

### 2.2. Patients

Between April 2013 and March 2024, 598 patients underwent surgical resection of histologically confirmed intracranial meningiomas at our institution. Among them, 146 consecutive patients (52 men and 94 women) with skull base meningiomas directly contacting or compressing the optic nerve and/or optic chiasm were included. Patients were classified according to preoperative visual status as having normal visual function (*n* = 53; mean age, 59.4 years) or visual dysfunction (*n* = 93; mean age, 59.4 years), based on comprehensive ophthalmologic evaluation. Patients with normal visual function had no subjective visual complaints and completely normal ophthalmologic findings, whereas patients with any objective visual abnormality were classified as having visual dysfunction regardless of the presence or absence of subjective visual symptoms.

Among the 53 patients with normal preoperative visual function, the primary indication for surgery was incidental detection of a tumor contacting or compressing the anterior visual pathway in 28 patients (52.8%), documented tumor growth during follow-up in 15 (28.3%), and nonvisual symptoms, such as seizures or olfactory disturbance, in 10 (18.9%). In the latter group, ophthalmologic evaluation confirmed normal visual function despite tumor contact with or compression of the optic nerve or chiasm.

The present study was designed to evaluate postoperative visual outcomes according to preoperative visual status among surgically treated patients; therefore, patients managed conservatively were not included.

### 2.3. Exclusion Criteria

Optic nerve sheath meningiomas were excluded because their tumor origin and pattern of optic nerve encasement differ substantially from skull base meningiomas involving the anterior visual pathway and are associated with distinct visual prognoses and treatment strategies.

### 2.4. Imaging Evaluation

Optic canal involvement was assessed retrospectively on preoperative MRI in all patients and defined as intracanalicular tumor extension identifiable on heavily T2-weighted images. Preoperative imaging was re-reviewed to determine optic canal involvement according to the detailed anatomical tumor classification described below.

### 2.5. Surgical Procedures

Microscopic or exoscopic transcranial approaches (pterional, interhemispheric, or subfrontal) were primarily used, whereas selected recent cases underwent an endoscopic endonasal approach. Visual evoked potentials (VEPs) and electroretinography were monitored intraoperatively [17]. A ≥50% reduction in VEP amplitude prompted modification or temporary interruption of surgical manipulation [17]. Gross total resection was intended unless severe adhesion to the optic apparatus or sustained VEP deterioration required intentional residual tumor.

### 2.6. Data Collection and Variables

The following variables were analyzed: age, sex, tumor volume, tumor location, optic canal involvement, preoperative visual status, and postoperative visual function. For the statistical analyses, tumor location was categorized as medial or lateral to the optic nerve using the original classification applied in this study. This binary classification was retained as a parsimonious variable for the regression analyses and as a matching covariate in propensity score matching because subdivision into multiple anatomical categories would have resulted in small strata and increased model complexity. For detailed anatomical description, all tumors were additionally classified according to their attachment site as medial sphenoid wing, anterior clinoid, tuberculum sellae, posterior planum sphenoidale, or diaphragma sellae meningiomas. The indication for surgery was additionally recorded in patients with normal preoperative visual function. Tumor volume was estimated using the formula length × width × height ÷ 2.

### 2.7. Preoperative Ophthalmologic Evaluation and Classification of Visual Status

Comprehensive ophthalmologic evaluation included best-corrected visual acuity, visual field testing (Humphrey and/or Goldmann perimetry), and fundus examination. Humphrey or Goldmann perimetry was selected by the treating ophthalmologist according to the patient’s clinical condition and test feasibility as part of routine clinical care. Because these examinations do not provide directly interchangeable quantitative indices, visual-field findings were evaluated clinically rather than converted to a common numerical scale. Visual acuity impairment was defined as an abnormal reduction in best-corrected visual acuity considered attributable to dysfunction of the anterior visual pathway, after excluding ocular causes of reduced visual acuity. Visual field impairment was defined as any reproducible visual field defect or sensitivity reduction detected by Humphrey or Goldmann perimetry. Patients were classified as having normal visual function only when they had no subjective visual complaints and completely normal ophthalmologic findings, including visual acuity, visual fields, and funduscopic findings. Patients with any objective visual abnormality, including an isolated visual field defect without subjective symptoms, were classified as having visual dysfunction.

### 2.8. Assessment of Visual Function

For the present study, postoperative visual outcome was assessed at approximately 6 months after surgery using visual acuity and visual field examinations. Visual outcome was classified as improved if either visual acuity or visual field improved without worsening of the other, worsened if either parameter deteriorated, and unchanged if both remained stable. Because mild optic pathway dysfunction may manifest as visual field loss before affecting visual acuity, both parameters were weighted equally in determining overall visual outcome.

### 2.9. Endpoints

The primary endpoint was postoperative normal visual function. In patients with normal preoperative visual function, this was defined as preservation of completely normal preoperative visual function. In patients with preoperative visual dysfunction, it was defined as complete recovery to normal visual function, with normalization of both visual acuity and visual field findings. This state-based endpoint was selected to compare the probability of having normal visual function after surgery according to preoperative visual status, rather than to compare the magnitude of change from baseline.

The secondary endpoint was favorable visual outcome, defined as preservation of normal vision in patients with normal preoperative visual function and improvement of visual function, with or without complete recovery to normal visual function, in patients with preoperative visual dysfunction.

### 2.10. Statistical Analysis

Categorical variables were compared using Fisher’s exact test and continuous variables using the Mann–Whitney U test. Univariable and multivariable logistic regression analyses were performed in the full cohort to identify factors associated with postoperative visual outcomes. Variables with a *p* value < 0.20 in the univariable analyses were included in the multivariable logistic regression models. To reduce baseline differences between patients with and without preoperative visual dysfunction, PSM was performed among patients with complete data for all matching covariates. One patient with an en plaque tumor was excluded from PSM because tumor volume could not be reliably quantified. Propensity scores for normal preoperative visual function were estimated using logistic regression including age, sex, tumor volume, tumor laterality, and optic canal involvement. One-to-one nearest-neighbor matching without replacement was performed on the logit of the propensity score using a caliper width of 0.2 SD, with the closest available pairs prioritized. Patients who were not matched were excluded from the matched-pair outcome analysis but remained included in the full-cohort analyses. Covariate balance after matching was assessed using standardized mean differences (SMDs), with values < 0.1 indicating adequate balance [18,19]. Because PSM generated paired binary observations, McNemar tests were used to compare outcomes between matched pairs. The exact procedure was selected because of the small numbers of discordant pairs. Statistical analyses were conducted using JMP Pro version 18.2.1 (SAS Institute Inc., Cary, NC, USA) and R version 4.4.0. Two-sided *p* values < 0.05 were considered statistically significant.

### 2.11. Use of a Large Language Model

OpenAI ChatGPT(OpenAI, San Francisco, CA, USA; web version, https://chatgpt.com/, accessed on 14 October 2026) was used during manuscript preparation to assist with English-language editing, wording refinement, and organization of manuscript-related text. All AI-assisted text was critically reviewed and revised by the authors, who take full responsibility for the content of the manuscript. ChatGPT was not used to generate or analyze study data or to perform statistical analyses.

## 3. Results

### 3.1. Baseline Characteristics

A total of 146 patients were included (53 with normal preoperative visual function and 93 with preoperative visual dysfunction). Baseline characteristics are summarized in Table 1. Mean age and sex distribution were comparable between groups. Tumor laterality, using the medial/lateral classification applied for statistical analysis, also did not differ significantly. The median preoperative tumor volume was 10.1 [4.8–16.1] cm^3^ in patients with normal preoperative visual function and 10.3 [3.6–30.4] cm^3^ in those with preoperative visual dysfunction (*p* = 0.326); however, the SMD was 0.40, indicating baseline imbalance. Optic canal involvement was significantly more frequent in patients with preoperative visual dysfunction than in those with normal preoperative visual function (45.2% vs. 15.1%, *p* < 0.001).

**Figure 1:**
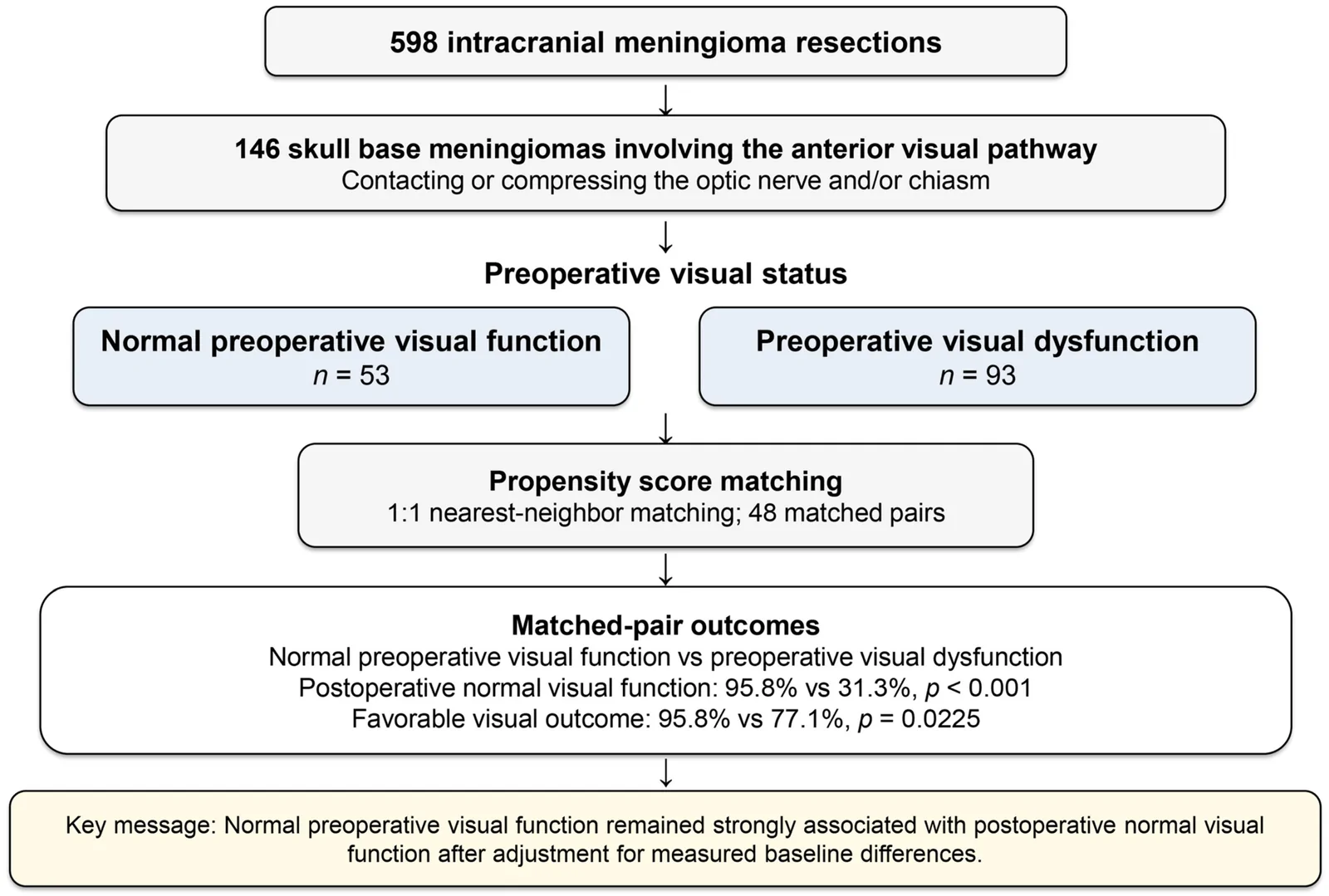
Study schema and principal findings. Of 598 intracranial meningioma resections, 146 patients with skull base meningiomas involving the anterior visual pathway were included. Patients were classified according to preoperative visual status as having normal preoperative visual function (*n* = 53) or preoperative visual dysfunction (*n* = 93). Propensity score matching yielded 48 matched pairs. In the matched cohort, postoperative normal visual function was observed in 95.8% and 31.3% of patients, respectively, and favorable visual outcome in 95.8% and 77.1%, respectively.

An overview of the study cohort, preoperative visual-status groups, propensity score matching, and principal matched-pair outcomes is shown in Figure 1.

Detailed anatomical tumor distribution and optic canal involvement are shown in Table 2A. In the normal preoperative visual function group, tumors were classified as medial sphenoid wing in 14 patients, anterior clinoid in 11, tuberculum sellae in 13, posterior planum sphenoidale in 15, and diaphragma sellae in none. Corresponding numbers in the preoperative visual dysfunction group were 15, 29, 31, 15, and 3, respectively. Among the 93 patients with preoperative visual dysfunction, 39 (41.9%) had visual field impairment without visual acuity impairment, whereas 54 (58.1%) had impairment of both visual acuity and visual field; no patient had isolated visual acuity impairment (Table 2B).

Among the 53 patients with normal preoperative visual function, the primary indication for surgery was incidental detection of a tumor contacting or compressing the anterior visual pathway in 28 patients (52.8%), documented tumor growth during follow-up in 15 (28.3%), and nonvisual symptoms, such as seizures or olfactory disturbance, in 10 (18.9%).

### 3.2. Postoperative Visual Outcomes

At approximately 6 months after surgery, normal visual function was preserved in 51 of 53 patients (96.2%) with normal preoperative visual function, whereas 2 (3.8%) experienced postoperative visual deterioration. Among 93 patients with preoperative visual dysfunction, 65 (69.9%) improved, including 26 (28.0% of the group; 40.0% of those who improved) who achieved complete recovery to normal visual function, while 4 (4.3%) worsened. Accordingly, the primary endpoint was achieved in 96.2% of patients with normal preoperative visual function and 28.0% of those with preoperative visual dysfunction (*p* < 0.001), whereas the secondary endpoint was achieved in 96.2% and 69.9%, respectively (*p* < 0.001; Table 3).

Among the 53 patients with normal preoperative visual function, 2 patients (3.8%) experienced postoperative visual deterioration. One patient developed a mild peripheral visual field deficit without loss of visual acuity, whereas the other experienced deterioration in both visual acuity and visual field. Intraoperative events suggested that these deficits were related to surgical manipulation involving the optic nerve in one patient and a perforating branch of the superior hypophyseal artery in the other. Among the 93 patients with preoperative visual dysfunction, 4 patients (4.3%) experienced postoperative worsening; 2 had worsening of visual field findings without deterioration in visual acuity, whereas 2 had worsening of both visual acuity and visual field findings.

### 3.3. Univariable and Multivariable Logistic Regression Analyses

Results of the univariable and multivariable logistic regression analyses are summarized in Table 4. Normal preoperative visual function was strongly associated with both postoperative normal visual function and favorable visual outcome.

For the primary endpoint, normal preoperative visual function was strongly associated with postoperative normal visual function in univariable analysis (OR 65.71, 95% CI 18.55–420.00, *p* < 0.001), whereas tumor volume was not (*p* = 0.186). Absence of optic canal involvement was also significantly associated with normal visual function (OR 4.91, 95% CI 2.37–10.63, *p* < 0.001).

For the secondary endpoint, normal preoperative visual function was significantly associated with favorable visual outcome (OR = 10.98, 95% CI 3.10–70.00, *p* < 0.001). Tumor volume (OR per 1 cm^3^ 1.03, 95% CI 1.01–1.08, *p* = 0.014) and absence of optic canal involvement (OR 4.74, 95% CI 2.06–11.39, *p* < 0.001) were also significantly associated with favorable visual outcome.

Variables with a *p* value < 0.20 in the univariable analyses were included in the multivariable models. For the primary endpoint, normal preoperative visual function remained independently associated with postoperative normal visual function (adjusted OR 54.89, 95% CI 15.07–356.08, *p* < 0.001). Absence of optic canal involvement was also independently associated with postoperative normal visual function (adjusted OR 3.13, 95% CI 1.24–8.48, *p* = 0.015), whereas tumor volume was not significantly associated with postoperative normal visual function (adjusted OR 1.00 per 1 cm^3^, 95% CI 0.98–1.02, *p* = 0.951). For the secondary endpoint, normal preoperative visual function (adjusted OR 10.24, 95% CI 2.72–67.06, *p* < 0.001), tumor volume (adjusted OR per 1 cm^3^ 1.05, 95% CI 1.02–1.09, *p* = 0.002), and absence of optic canal involvement (adjusted OR 3.49, 95% CI 1.39–9.18, *p* = 0.0077) were independently associated with favorable visual outcome.

### 3.4. Propensity Score-Matched Analysis

**Figure 2:**
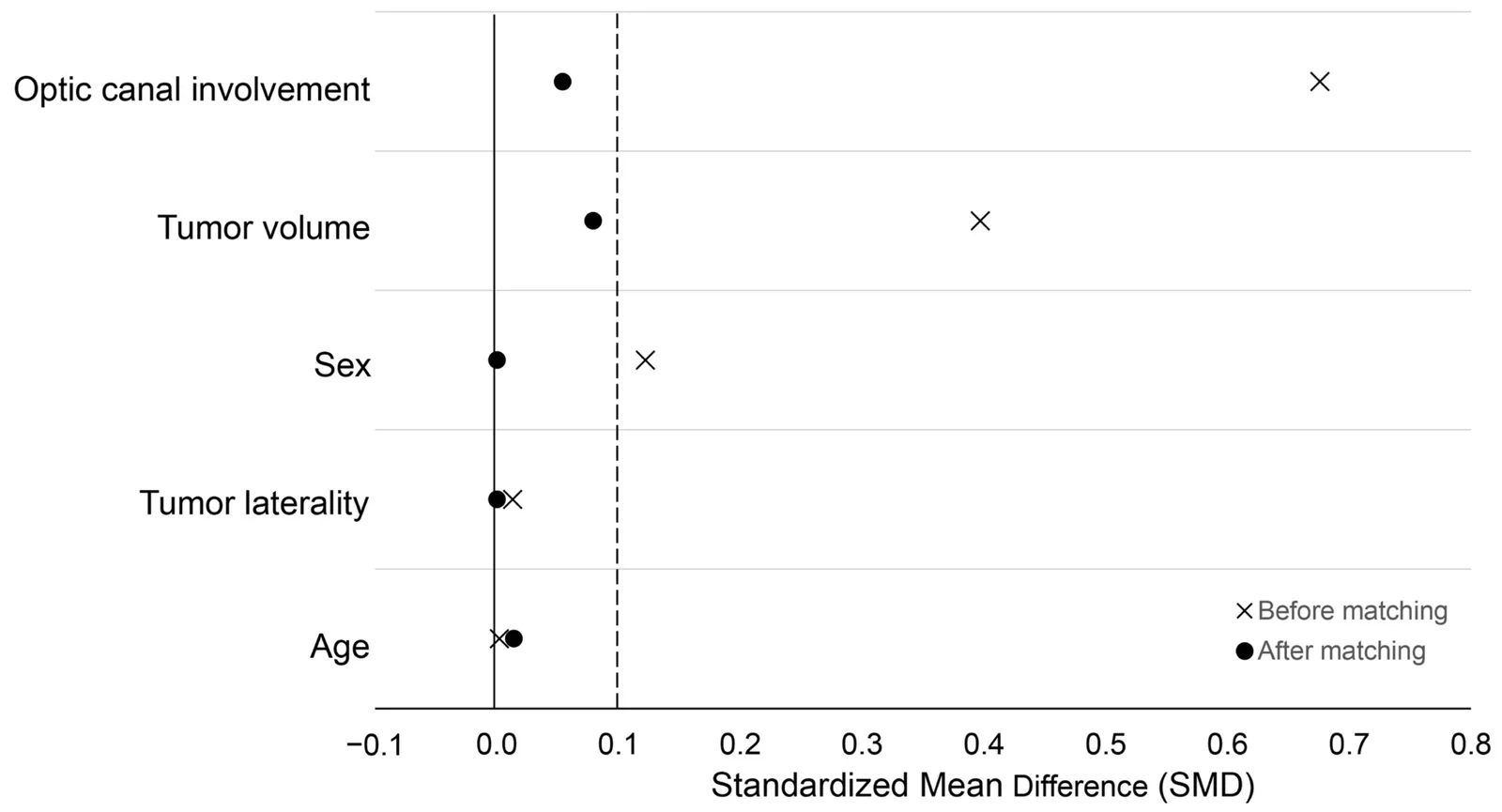
Covariate balance before and after propensity score matching. Crosses indicate standardized mean differences (SMDs) before matching, and filled circles indicate SMDs after matching. The solid vertical line indicates an SMD of 0, and the dashed vertical line represents the conventional threshold of an SMD of 0.1.

Propensity score matching was performed in 145 patients with complete data for all matching covariates; one patient with preoperative visual dysfunction was excluded because tumor volume could not be reliably quantified in an en plaque tumor. After PSM, 48 matched pairs were obtained, with adequate balance across all matching covariates (all SMDs < 0.1; Figure 2, Table S1).

Postoperative normal visual function was preserved or achieved in 95.8% (46/48) of patients with normal preoperative visual function compared with 31.3% (15/48) of those with preoperative visual dysfunction. Among the 48 matched pairs, 32 pairs were discordant, with postoperative normal visual function observed only in the patient with normal preoperative visual function, whereas one pair showed the opposite pattern (exact McNemar *p* < 0.001; Table S2).

Favorable visual outcome was observed in 95.8% (46/48) of patients with normal preoperative visual function compared with 77.1% (37/48) of those with preoperative visual dysfunction. Among the 48 matched pairs, 11 pairs were discordant, with a favorable visual outcome observed only in the patient with normal preoperative visual function, whereas two pairs showed the opposite pattern (exact McNemar *p* = 0.0225; Table S3).

## 4. Discussion

### 4.1. Principal Findings and Clinical Context

The principal finding of this study is not simply that patients with normal preoperative vision had better postoperative visual function than patients with established visual impairment. Rather, among skull base meningiomas directly involving the anterior visual pathway, the likelihood of maintaining or achieving normal vision differed substantially according to preoperative visual status, and this association persisted after adjustment for relevant baseline clinical and anatomical differences.

This distinction is clinically important because management recommendations for incidental asymptomatic meningiomas generally favor observation with serial imaging [2,3,4,5,6]. These recommendations are based largely on cohorts combining tumors from heterogeneous intracranial locations. The anterior visual pathway, however, represents an anatomically constrained and functionally vulnerable region in which relatively limited tumor growth may result in clinically important and potentially irreversible visual dysfunction. Our findings therefore address a different clinical question from whether asymptomatic meningiomas in general should undergo prophylactic surgery: whether tumors already contacting or compressing the optic nerves or chiasm warrant separate consideration when determining surgical timing.

In the present cohort, preoperative visual status was consistently strongly associated with postoperative visual outcomes across univariable, multivariable, and propensity score-matched analyses. Importantly, patients with preoperative visual dysfunction had a substantially higher prevalence of optic canal involvement, and tumor volume also showed baseline imbalance. After these and other measured differences were balanced by PSM, the association between preoperative visual status and postoperative visual outcomes remained marked. These findings suggest that the observed differences were not explained solely by the measured demographic and anatomical differences between the two groups.

Tumor volume showed different associations according to the visual endpoint examined. It was not independently associated with postoperative normal visual function in the primary analysis, whereas larger tumor volume was associated with the secondary favorable visual outcome. This latter finding should be interpreted cautiously because the secondary endpoint differs conceptually from the primary endpoint: it combines preservation of normal visual function in patients with normal preoperative vision with any postoperative improvement, including partial improvement, in patients with preoperative visual dysfunction. Accordingly, the association between tumor volume and the secondary endpoint should not be interpreted as indicating that larger tumors are more likely to achieve complete postoperative visual normalization.

### 4.2. Relation to Prior Evidence and Guidelines

Large observational studies of incidental meningiomas generally support initial observation (“watch-and-scan”) [2,3,4,5,6]. However, these studies pool tumors from heterogeneous locations and rarely isolate lesions directly involving the optic nerves or chiasm. Natural-history studies have also demonstrated location-dependent differences in tumor behavior, further supporting the need to consider tumor location when interpreting data from heterogeneous incidental meningioma cohorts [20]. Their findings therefore may not be fully generalizable to meningiomas involving the anterior visual pathway. The EANO guideline likewise endorses surveillance for many asymptomatic meningiomas while emphasizing preservation of cranial nerve function in skull base tumors and acknowledging the limited availability of location-specific controlled data [21].

Only a few studies have specifically addressed asymptomatic meningiomas involving the anterior visual pathway. Sturiale et al. [16] reported favorable postoperative visual outcomes in asymptomatic patients, but adjusted comparisons between asymptomatic and symptomatic patients were not performed. The present study extends these observations by accounting for baseline differences using both multivariable analysis and PSM, including anatomical factors directly relevant to visual prognosis such as optic canal involvement.

Site-specific surgical series have consistently identified preoperative visual status and symptom duration as important determinants of postoperative visual recovery [13,15,22,23]. Optic canal involvement has also been associated with visual impairment and postoperative visual outcome in tuberculum sellae and clinoidal meningiomas [14,24,25,26]. Contemporary comparative and multicenter studies have further characterized visual outcomes across different surgical strategies for tuberculum sellae and other anterior skull-base meningiomas [27,28,29]. A more recent study using detailed quantitative assessment of visual impairment further emphasized the importance of preoperative visual function in postoperative visual outcome [30]. These observations support the biological and clinical plausibility of our findings. However, previous studies have primarily focused on predictors of recovery among patients with visual dysfunction rather than on the preservation of normal visual function in patients undergoing surgery before visual dysfunction becomes established.

### 4.3. Clinical Implications

The present findings should not be interpreted as supporting prophylactic surgery for all incidentally detected asymptomatic meningiomas. Studies of surgically treated asymptomatic or incidental meningiomas likewise underscore the importance of careful patient selection rather than routine surgical intervention [31,32]. Rather, our findings suggest that tumor location and its relationship to critical neural structures should be incorporated into decisions regarding observation versus intervention.

In this surgically treated cohort, patients with preoperative visual dysfunction were less likely to have normal postoperative visual function than those with normal preoperative visual function. This consideration may be particularly relevant when the optic canal or other anatomically constrained portions of the anterior visual pathway are involved, where even limited tumor extension may compromise visual function [24].

Importantly, surgery in patients with normal preoperative visual function was not risk-free. Two of 53 patients (3.8%) developed postoperative visual deterioration. In one patient, a mild peripheral visual field deficit occurred without deterioration in visual acuity and was considered related to manipulation of the optic nerve. In the other, both visual acuity and visual field worsened, likely in association with manipulation of a perforating branch of the superior hypophyseal artery. These cases illustrate that visual injury may occur through either direct manipulation of the optic apparatus or compromise of its vascular supply, even when intraoperative monitoring and risk-reduction strategies are used. Therefore, the potential benefit of operating before visual dysfunction develops must be weighed against this non-zero surgical risk. From an ethical perspective, our findings should therefore not be interpreted as supporting routine prophylactic surgery in all patients with normal visual function. Rather, this non-zero surgical risk should be incorporated into individualized decision-making.

Accordingly, meningiomas involving the anterior visual pathway may warrant separate consideration from asymptomatic meningiomas at other intracranial locations. Surgical decisions should remain individualized and should incorporate tumor growth, patient age and comorbidities, surgical risk, anatomical characteristics, and patient preference. The present findings indicate that preoperative visual status is an important factor to consider in this decision-making process, but they do not establish that earlier surgery improves visual outcomes or define an optimal surgical timing strategy.

### 4.4. Methodological Strengths

This study incorporated two complementary approaches to address confounding inherent in retrospective comparisons. Multivariable logistic regression identified factors independently associated with postoperative visual outcomes, while PSM created groups balanced for age, sex, tumor volume, tumor laterality, and optic canal involvement. Covariate balance was verified using SMDs rather than relying solely on statistical significance testing.

We also used clinically defined visual endpoints based on comprehensive ophthalmologic evaluation. The primary endpoint was intentionally defined as a common postoperative state—normal visual function—rather than as a common change-score endpoint. Thus, patients with normal preoperative visual function could meet the endpoint by preserving normal vision, whereas those with preoperative visual dysfunction could meet it only by complete recovery to normal vision. This design allowed comparison of the probability of being visually normal after surgery according to preoperative visual status. The secondary endpoint complemented this analysis by capturing favorable visual change, including partial improvement.

### 4.5. Limitations

Several limitations warrant consideration.

First, this was a retrospective single-center study, and selection bias and residual confounding may remain despite consecutive case inclusion, multivariable adjustment, and PSM. In addition, the single-center setting may limit the generalizability of our findings to other institutions with different patient populations, surgical indications, or treatment strategies. Residual confounding may also have resulted from factors not incorporated into the matching model, including tumor consistency, surgical approach, degree of adhesion to the optic apparatus, temporal changes in surgical technique and surgeon experience, and, among patients with preoperative visual dysfunction, the duration and severity of visual impairment. The findings therefore demonstrate association rather than causation. Second, the study included only surgically treated patients, and surgical selection among patients with normal preoperative visual function was not randomized and reflected heterogeneous clinical indications, including tumor contact or compression of the anterior visual pathway, documented tumor growth, and nonvisual symptoms. The absence of a conservatively managed comparison group introduces selection bias and limits any direct comparison between surgery and observation. The results should therefore not be interpreted as establishing surgery as preferable to observation for all tumors involving the anterior visual pathway in patients with normal visual function. Third, because preoperative visual status was the principal exposure of interest and was intrinsically related to the definition of postoperative normal visual function, the magnitude of its association with the primary endpoint should be interpreted cautiously. PSM addresses measured baseline differences but cannot eliminate this inherent relationship between preoperative status and the endpoint. Fourth, visual-field testing was not uniform (Humphrey and/or Goldmann perimetry), and directly comparable quantitative visual-field indices and optical coherence tomography parameters were not consistently available because of the retrospective design. We therefore relied on clinically documented visual-field normality, improvement, stability, or deterioration rather than converting heterogeneous examinations into a nonvalidated common numerical scale. Optical coherence tomography parameters were also not consistently available. In addition, the degree of optic nerve or chiasmal compression was not assessed using a standardized quantitative grading system in this retrospective cohort. The present analysis focused on visual outcomes at approximately 6 months after surgery and did not evaluate long-term changes in visual function. Finally, the matched sample consisted of only 48 pairs, limiting statistical precision, particularly for secondary analyses, and precluding robust subgroup analyses within the matched cohort. A randomized trial evaluating surgical timing in patients with normal visual function whose tumors already contact or compress the optic nerves or chiasm would be difficult to conduct because of the potential risk of irreversible visual deterioration during observation. Multicenter prospective registries using standardized ophthalmologic and imaging assessments may therefore provide a more feasible approach to validating these findings.

## 5. Conclusions

Among surgically treated patients with skull base meningiomas involving the anterior visual pathway, normal preoperative visual function was strongly associated with postoperative normal visual function after adjustment for measured clinical and anatomical differences. Because this retrospective study included only surgically treated patients and lacked a conservatively managed comparison group, the findings do not establish that earlier surgery improves visual outcomes or define an optimal surgical timing strategy. Rather, they suggest that preoperative visual status and the anatomical relationship of the tumor to the anterior visual pathway should be incorporated into individualized decisions regarding surveillance and surgical intervention.

## Figures and Tables

**Table 1:** Baseline characteristics of the study population.

Variable	Normal Preoperative Visual Function *n* = 53	Preoperative Visual Dysfunction *n* = 93	*p* Value
Age, mean ± SD	59.4 ± 10.2	59.4 ± 12.0	0.990
Sex, female, *n* (%)	32 (60.4)	62 (66.7)	0.476
Tumor laterality, *n* (%)	Medial 28 (52.8), Lateral 25 (47.2)	Medial 48 (51.6), Lateral 45 (48.4)	1.000
Tumor volume (cm^3^), median (IQR)	10.1 [4.8–16.1], range 0.4–70.3	10.3 [3.6–30.4], range 0.9–124.6	0.326
Optic canal involvement, *n* (%)	8 (15.1)	42 (45.2)	<0.001

Age is presented as mean ± standard deviation (SD), tumor volume as median [interquartile range (IQR)] and range, and categorical variables as number (%). *p*-values were calculated using Fisher’s exact test or the Mann–Whitney U test, as appropriate.

**Table 2:** (**A**) Detailed anatomical distribution and optic canal involvement according to preoperative visual status. (**B**) Pattern of preoperative visual dysfunction according to tumor location.

(**A**)				
**Tumor Location**	**Normal Visual Function, *n***	**Optic Canal Involvement, *n* (%)**	**Visual Dysfunction, *n***	**Optic Canal Involvement, *n* (%)**
Medial sphenoid wing	14	1 (7.1)	15	7 (46.7)
Anterior clinoid	11	2 (18.2)	29	19 (65.6)
Tuberculum sellae	13	3 (23.1)	31	11 (35.5)
Posterior planum sphenoidale	15	2 (13.3)	15	3 (20.0)
Diaphragma sellae	0	–	3	2 (66.7)
Total	53	8 (15.1)	93	42 (45.2)
(**B**)				
**Tumor Location**	***n***	**Visual Acuity Loss Only, *n* (%)**	**Visual Field Disturbance Only, *n* (%)**	**Both, *n* (%)**
Medial sphenoid wing	15	0 (0.0)	5 (33.3)	10 (66.7)
Anterior clinoid	29	0 (0.0)	14 (48.3)	15 (51.7)
Tuberculum sellae	31	0 (0.0)	14 (45.2)	17 (54.8)
Posterior planum sphenoidale	15	0 (0.0)	4 (26.7)	11 (73.3)
Diaphragma sellae	3	0 (0.0)	2 (66.7)	1 (33.3)
Total	93	0 (0.0)	39 (41.9)	54 (58.1)

(**A**) Data are presented as number of patients and number (%) with optic canal involvement within each tumor-location category. Optic canal involvement was assessed on preoperative MRI and defined as intracanalicular tumor extension. Percentages were calculated separately within each preoperative visual-status group and tumor-location category. A dash indicates that no patient was included in that category. (**B**) Only patients with preoperative visual dysfunction are included. Visual dysfunction was categorized as visual acuity impairment only, visual field impairment only, or impairment of both visual acuity and visual field, irrespective of severity. Percentages were calculated within each tumor-location category.

**Table 3:** Postoperative visual outcomes at approximately 6 months.

Visual Outcome	Normal Preoperative Visual Function *n* = 53	Preoperative Visual Dysfunction *n* = 93	*p* Value
Normal visual function (primary endpoint) (%)	96.2	28.0	<0.001
Favorable visual outcome (secondary endpoint) (%)	96.2	69.9	<0.001
Worsened (%)	3.8	4.3	1.000

Data are presented as proportions of patients in each group. Normal visual function denotes preservation of completely normal vision in patients with normal preoperative visual function and complete recovery to normal vision in patients with preoperative visual dysfunction, whereas favorable visual outcome denotes preservation of normal vision in patients with normal preoperative visual function and improvement in visual function, with or without complete recovery, in patients with preoperative visual dysfunction. *p*-values were calculated using Fisher’s exact test.

**Table 4:** Univariable and multivariable logistic regression analyses for visual outcomes.

Variable	Primary Endpoint (Normal Visual Function) Univariable OR (95% CI), *p*	Primary Endpoint Multivariable aOR (95% CI), *p*	Secondary Endpoint (Favorable Visual Outcome) Univariable OR (95% CI), *p*	Secondary Endpoint Multivariable aOR (95% CI), *p*
Preoperative visual status (normal vs. visual dysfunction)	65.71 (18.55–420.00), <0.001	54.89 (15.07–356.08), <0.001	10.98 (3.10–70.00), <0.001	10.24 (2.72–67.06), <0.001
Optic canal involvement (absent)	4.91 (2.37–10.63), <0.001	3.13 (1.24–8.48), 0.015	4.74 (2.06–11.39), <0.001	3.49 (1.39–9.18), 0.0077
Tumor volume (per 1 cm^3^)	0.99 (0.97–1.01), 0.186	1.00 (0.98–1.02), 0.951	1.03 (1.01–1.08), 0.014	1.05 (1.02–1.09), 0.002
Age	1.00 (0.97–1.03), 0.900	–	1.01 (0.97–1.04), 0.737	–
Sex (male/female)	1.37 (0.69–2.72), 0.372	–	1.37 (0.59–3.41), 0.467	–
Tumor laterality (medial/lateral)	1.11 (0.58–2.12), 0.761	–	0.79 (0.34–1.77), 0.571	–

Data are presented as odds ratios (OR) from univariable analysis and adjusted odds ratios (aOR) from multivariable analysis, with 95% confidence intervals (CI) and *p*-values. Outcomes analyzed were postoperative normal visual function and favorable visual outcome.

## Data Availability

The de-identified data underlying this article are available from the corresponding author upon reasonable request, subject to institutional and ethical requirements.

## Supplementary Materials

The following supporting information can be downloaded at: https://www.mdpi.com/article/10.3390/cancers18203324/s1, Table S1: Covariate balance before and after propensity score matching; Table S2: Matched-pair cross-tabulation of postoperative normal visual function after propensity score matching; Table S3: Matched-pair cross-tabulation of favorable visual outcome after propensity score matching.
