# Supplementary material for: Surgical Timing in Relation to Visual Dysfunction in Skull Base Meningiomas Involving the Anterior Visual Pathway: A Propensity Score-Matched Analysis
Source: Cancers (Basel). 2026 Oct 15;18(20):3324. doi: 10.3390/cancers18203324 (PMC13702277; doi:10.3390/cancers18203324)
Supplement: Supplementary file 1 [file cancers-18-03324-s001.zip › Tables S1-S3.pdf]

Table S1. Covariate balance before and after propensity score matching.

Covariate	SMD (Before matching)	SMD (After matching)
Optic canal involvement	0.676	0.054
Tumor volume	0.397	0.079
Sex	0.122	0.000
Tumor laterality	0.013	0.000
Age	0.002	0.014

Table Legend

Standardized mean differences (SMDs) for baseline covariates before and after propensity score matching. Adequate covariate balance was defined as an absolute SMD < 0.1.

Table S2. Matched-pair cross-tabulation of postoperative normal visual function after propensity score matching.

	Matched patient with preoperative visual dysfunction		
Patient with normal preoperative visual function	Normal visual function	Not normal visual function	Total
Normal visual function	14	32	46
Not normal visual function	1	1	2
Total	15	33	48

Table Legend

Data represent numbers of matched pairs. Among the 48 matched pairs, 32 were discordant with postoperative normal visual function observed only in the patient with normal preoperative visual function, whereas one pair showed the opposite pattern. The difference was significant by the two-sided exact McNemar test ($p<0.001$).

Table S3. Matched-pair cross-tabulation of favorable visual outcome after propensity score matching.

	Matched patient with preoperative visual dysfunction		
Patient with normal preoperative visual function	Favorable visual outcome	Not favorable visual outcome	Total
Favorable visual outcome	35	11	46
Not favorable visual outcome	2	0	2
Total	37	11	48

Table Legend

Data represent numbers of matched pairs. Among the 48 matched pairs, 11 were discordant with a favorable visual outcome observed only in the patient with normal preoperative visual function, whereas two pairs showed the opposite pattern. The difference was significant by the two-sided exact McNemar test ($p=0.0225$).
